# Integrated surgical intervention for intradural extramedullary hemangioblastoma of the cervical spine: a case report and literature review

**DOI:** 10.3389/fonc.2024.1387005

**Published:** 2024-11-12

**Authors:** Yao Wang, Qing Zhu, Ailin Chen, Chungang Dai, Longjiang Xu, Minfeng Sheng, Qiang Huang, Qing Lan, Qingchun Mu, Rujun Li

**Affiliations:** ^1^ Department of Neurosurgery, Second Affiliated Hospital of Soochow University, Suzhou, Jiangsu, China; ^2^ Department of Pathology, Second Affiliated Hospital of Soochow University, Suzhou, Jiangsu, China

**Keywords:** spinal hemangioblastoma, intradural extramedullary, Von Hippel-Lindau syndrome, rare diseases, integrated surgical intervention, case report

## Abstract

**Introduction:**

The incidence of hemangioblastoma is low, constituting only 1-5% of all spinal cord tumors. Specifically, intradural extramedullary hemangioblastoma without Von Hippel-Lindau syndrome represents an exceedingly rare condition.

**Methods:**

We report the first documented case of cervical intradural extramedullary hemangioblastoma in China. A 53-year-old male patient presented with a 3-year history of mild right hemiplegia, segmental muscle strength and sensation impairment, and a positive pyramidal tract sign. MRI showed an abnormal oval signal focus in the intradural and extramedullary region at the C6-C7 vertebral level. Before surgery, angiography was performed to identify the supplying arteries and draining veins. Subsequent interventional therapy achieved over 90% occlusion of blood vessels, creating optimal conditions for complete resection of the spinal tumor.

**Results:**

The patient demonstrated satisfactory postoperative recovery with significant restoration of sensory and motor functions. Pathological examination showed a significant upregulation of CD31 in tumor cells and a substantial presence of the neuro-specific marker S100 in the tumor stroma, consistent with the diagnostic criteria for spinal hemangioblastoma.

**Conclusion:**

The rarity of cervical intradural extramedullary hemangioblastoma without Von Hippel-Lindau syndrome was reaffirmed by a comprehensive review of the existing literature. Complete tumor resection remains the optimal approach for managing this uncommon condition, generally resulting in a favorable prognosis. Traditional open fenestration surgery is linked to elevated risks of bleeding and trauma. Meanwhile, endovascular injection of embolic agents may lead to residual lesions and an increased risk of recurrence. Therefore, we recommend a one-time combined treatment conducted in a hybrid operating room to achieve complete resection and effectively reduce intraoperative bleeding risk. Despite presenting challenges and requiring high proficiency, we still recommend this type of combined surgery as a suitable therapeutic option for such diseases.

## 1 Introduction

Hemangioblastomas (HBs) are rare, low-grade benign tumors, representing 1-3% of central nervous system (CNS) tumors (1). Of these, approximately 70-80% are found in the cerebellar hemisphere, 10-15% in the vermis, 10% in the brainstem, and only 3.2% in the spinal cord, where they account for 1–5% of intramedullary spinal tumors in surgical series (1–4). However, the spinal hemangioblastoma is characterized by its slow growth and high vascularity, making up only 2-15% of primary malignant tumors in the spinal cord (5). The intramedullary form accounts for 41 to 84% of spinal HBs, while both intramedullary and extramedullary forms are observed in 11 to 37%, and only 3 to 22% present as purely extramedullary lesions. Despite the known endothelial cell origin of HBs, their incidental extramedullary pathogenesis remains unclear (6). Additionally, HBs are also associated with Von Hippel-Lindau (VHL) syndrome, which is caused by mutations in the VHL gene located on chromosome 3p25-26 (7). At the same time, compared with patients with sporadic HBs (20.5%), patients with intradural extramedullary (IDEM) HBs associated with VHL syndrome (88.2%) were more common (8, 9). Therefore, IDEM spinal HBs without VHL syndrome are extremely rare. In this article, we will introduce the compound treatment plan for such rare diseases launched by our center, and discuss the specific diagnosis and treatment methods combined with the literature.

## 2 Case presentation

### 2.1 History

The patient, a 53-year-old man, presented with unexplained neck discomfort that started 3 years ago. He also experienced numbness and weakness in his right limb as well as difficulty walking. Physical examination revealed mild sensory segmental impairment and positive pyramidal signs. The patient had a history of hypertension and suffered from cerebral infarction 3 years ago, for which he had been taking orally administered enteric-coated aspirin tablets for an extended period of time. This patient did not have a family history of spinal HBs. Clinical evaluation for VHL syndrome did not reveal any retinopathy. Imaging studies of the chest, abdomen, head, and pelvis showed no evidence of other lesions. The patient had no relevant medical treatment history.

### 2.2 Imaging

**Figure 1:**
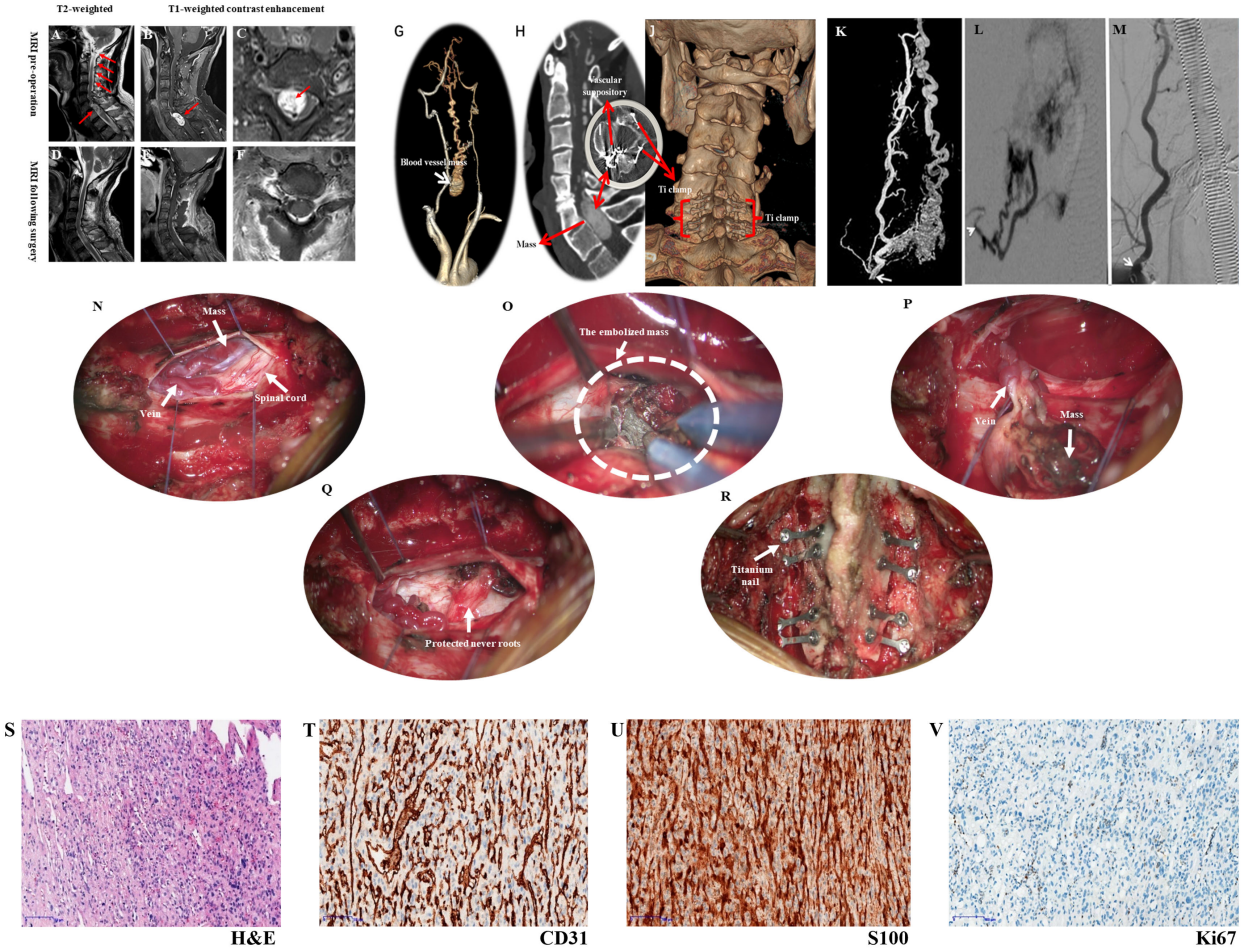
Presents the imaging examination, surgical procedure, and pathological findings. **Figure 1** **(A)** MRI shows a mass at the C6 and C7 levels, along with a beaded vascular flow shadow on the dorsal side of the spinal cord above it; **(B, C)** MRI reveals oval abnormal signal foci in the spinal canal at the level of C6-7 vertebral bodies, measuring approximately 16mm×11mm×26mm, exhibiting evident enhancement, as well as small patchy vascular flow shadows (red arrows) and changes indicative of spinal cord compression; **(D–F)** Depict images from postoperative period illustrating complete disappearance of the mass when compared to **(A–C)**; **(G)** The CTA reveals a vascular mass located between the bilateral vertebral arteries, potentially supplied by the superior thyroid artery; **(H)** Preoperative CT scan exhibits a rounded mass with lightly increased signal intensity within the spinal canal at C6-7; **(I)** Postoperative CT demonstrates complete disappearance of the mass, revealing residual shadows of the embolic agent; **(J)** Three-dimensional CT reconstruction confirms satisfactory fixation of vertebral plates in laminoplasty using titanium nails. **(K)** Selective angiography involved injecting a contrast agent through the distal end of the 4F VER catheter, entering the opening of the right thyroid neck trunk (white arrow). This depicted contrast agent entry into the tumor via the intervertebral foramen, causing hyperstaining and backflow towards the medulla oblongata; **(L)** The Echelon10 microcatheter was super selected into a branch of the tumor feeding artery from the superior thyroid artery (white arrow), resulting in tumor staining after contrast injection; **(M)** Following Onyx18 injection, angiography of the right thyrocervical trunk displayed well-developed main trunk arteries (white arrow) with nearly complete absence of tumor staining. **(N)** The dissection of the dura allows for the observation of varicose veins, tumors, and compression of the spinal cord; **(O)** After cutting the arachnoid membrane and flowing out the cerebrospinal fluid, the embolized tumor can be seen on the lateral side of the spinal cord; **(P)** The tumor has been dissociated and turned out of the tumor cavity; **(Q)** The nerve root is completely preserved; **(R)**. Reposition and titanium needle fixation of the lamina fix the vertebral plate, and maintain the stability of the spine. Pathological findings (×100 magnification) observed under a light microscope, **(S)** H&E staining revealed numerous fusiform tumor cells with irregular lumens and thin-walled blood vessels; **(T)** CD31 staining showed prominent vascular endothelial cells, with the presence of numerous irregular thin-walled blood vessels and some anastomosed blood vessels; **(U)** S100 expression was strongly positive in tumor cells; **(V)** Ki67 expression indicated a low tumor growth index of approximately 1%.

The MRI imaging of the neck revealed a space-occupying lesion at the C6-C7 level, causing compression on the spinal cord and exhibiting evident enhancement effect (**Figures 1A–C**). Within and above the lesion, empty vascular shadows were observed in the dorsal spinal cord. A vascular mass was identified between both sides of the vertebral arteries on CTA and CT, possibly receiving blood supply from the superior thyroid artery (**Figures 1G, H**). The findings on DSA were consistent with those on CTA (**Figure 1K**), indicating an arteriovenous malformation or spinal hemangioblastoma. The two types of diseases we have inferred are both benign, with a high probability of having a good prognosis.

### 2.3 Surgical intervention

The primary surgical approach for IDEM typically includes gross total resection (GTR) or subtotal resection (STR), optionally preceded by preoperative endovascular embolization or intraoperative venous drainage division (10–13). For tumors that cannot be fully resected, stereotactic radiotherapy and antiangiogenic therapy are viable alternatives (14–18). Preoperative endovascular embolization is an effective adjunct, reducing surgical bleeding and operative risks in selected cases (10–13). However, clear evidence supporting the benefits of preoperative endovascular embolization for improving tumor removal quality remains lacking (12). Considering the high likelihood of intraoperative bleeding and the necessity of complete tumor resection, we counseled the patient that right radial artery puncture cerebral angiography and embolization would be required prior to resection. This specific surgical procedure can be summarized as follows: “Puncture of the right radial artery for cerebral angiography, embolization of the tumor feeding artery, and resection of the tumor in the spinal canal under neuroelectrophysiological monitoring”. The patient was placed in the supine position after general anesthesia. Under the monitoring of the road map, the X-pedion10 microtubule guide wire shaped into a “J” shape at the tip was used to guide three Marathon microcatheters with straight tips to be placed into the three tumor feeding arteries (**Figure 1L**). The balloon was filled and a total of 2.5ml of Onyx18 glue was slowly injected. Angiography showed occlusion of more than 90% of the vessels in the vascular mass (**Figure 1M**). Then, the whole lamina and spinous process of C6-C7 were removed. The dura mater was cut and suspended, and a well-demarcated vascular mass with a size of 2.5cm×1.5cm×1.5cm filled with embolic agent was located at the right side of the spinal canal at C6 and C7 levels. The mass was located on the right side of the spinal cord, within the arachnoid, and originated from the region of the C6-C7 intervertebral foramen. Along the border of the mass, there was a gradual cessation of blood supply to the C6-C7 intervertebral foramen. The adhesion of the mass to the spinal cord and nerves was separated, and ultimately, the mass was completely removed. There was no evidence of residual tumor and the spinal cord remained intact. Subsequently, the removed C6 and C7 partial laminae combined with spinous process were reduced as a whole and fixed with 8 titanium connecting pieces and 16 titanium nails to consolidate the stability of the spine, and the wound was closed in a standard manner (**Figures 1N–R**). The intraoperative blood loss was only 300ml and the procedure lasted a total of 8 hours.

### 2.4 Postoperative course

**Figure 2:**
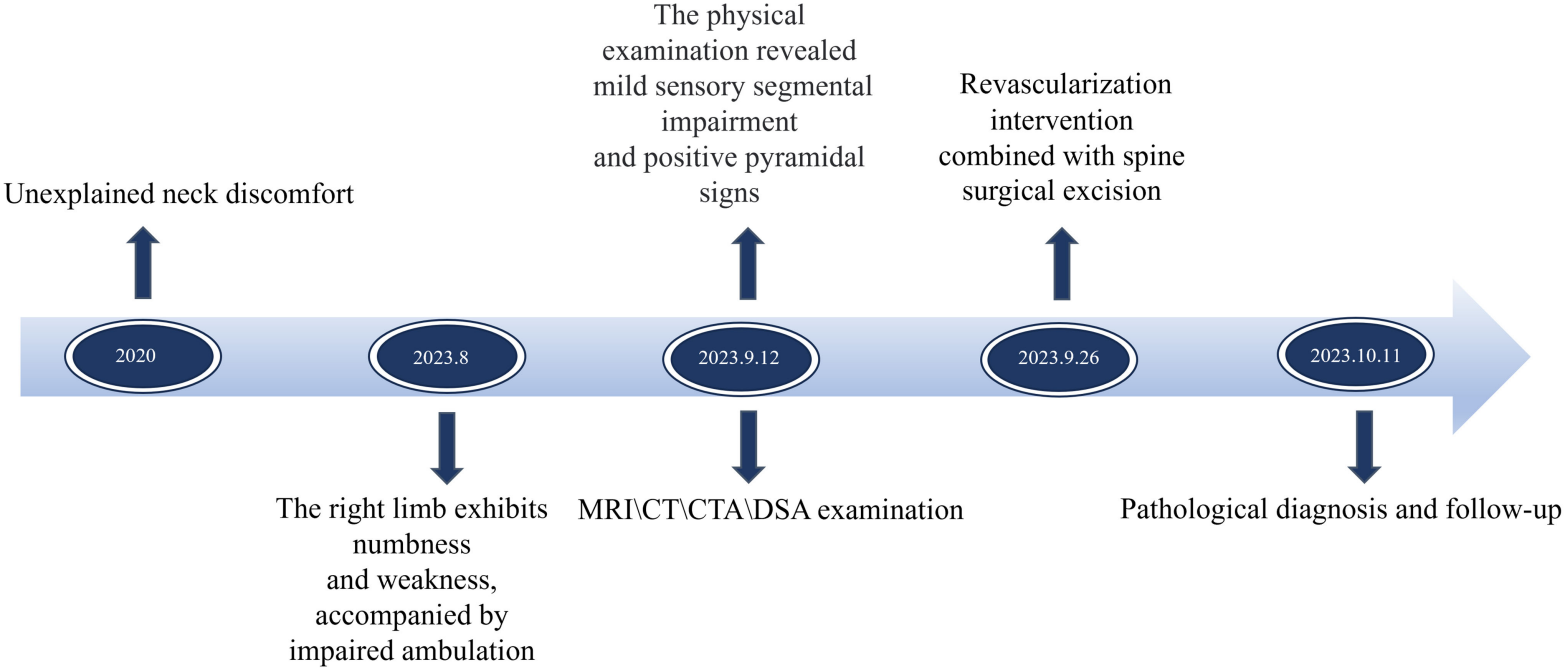
Presents a timeline of key points in the patient’s diagnosis and treatment.

Pathological examination of the resected tumor tissue was conducted post-surgery. The findings revealed that the tumor consisted of spindle-shaped tumor cells and blood sinuses. CD31 exhibited strong positivity, with immune complexes deposited irregularly on the vascular endothelium of the tumor, forming a grid-like pattern. S100 also demonstrated strong positivity, with immune complexes deposited on nerve bundles within the tumor stroma. These observations collectively supported the diagnosis of hemangioblastoma (**Figures 1S–V**). The postoperative recovery was satisfactory, as muscle strength returned to normal and sensory disturbances resolved. A follow-up MRI performed 9 days after surgery displayed effusion behind the lamina, edema in interspinous soft tissues, complete disappearance of mass shadow, and absence of residual lesions (**Figures 1D–F**). Postoperative CT scans show complete mass resolution, with only residual embolic agent shadows remaining (**Figure 1I**). Three-dimensional CT reconstruction further confirms successful vertebral plate fixation with titanium nails during laminoplasty (**Figure 1J**). During the postoperative follow-up for 6 months, the patient’s limb pain and numbness resolved, and normal gait. The Modified McCormick functional schema score (19) and Sensory pain scale score (19) decreased from 3 points before the operation to 1 point after the operation. The patient consistently adhered to the treatment regimen and did not experience any tolerability issues throughout the course of treatment. No significant complications were encountered. The timelines for crucial stages in patient diagnosis and care were meticulously devised (**Figure 2**).

## 3 Results

**Figure 3:**
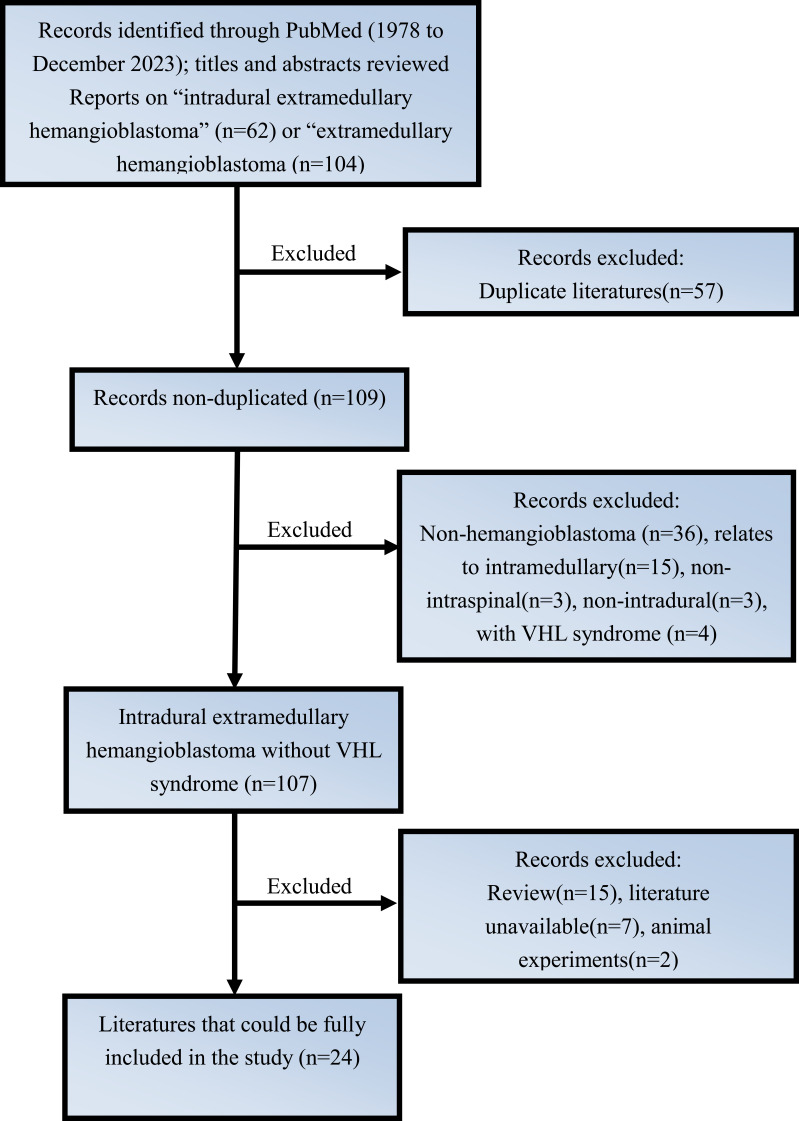
The final flowchart for literature enrollment.

We conducted a comprehensive literature review on this rare disease, covering the period from 1978 to 2023, and identified 27 cases of primary IDEM spinal HBs (3, 4, 6, 7, 14, 20–38). The criteria for including and excluding these literatures, along with specific patient information, are detailed in **Figure 3** and **Tables 1**, **2**. Of these cases, 12 were male (44.4%) and 15 were female (55.6%). The mean age at onset was 54.81 ± 15.33 years old, ranging from 17 to 82 years old. The tumors were most commonly located in the lumbar spine (n=7, 26%), followed by the cervical spine (n=6, 22%), thoracic spine (n=6, 22%), conus (n=3,11%), and cervicomedullary junction (n=2, 7%). What is more, the tumor also can span or accumulate more than one segment (n=3, 11%). The preoperative clinical manifestations of all patients can be summarized as follows: only sensory disorders (n=11, 41%), only motor disorders (n=2, 7%), combined sensory and motor disorders (n=13, 48%), and sensory and motor disorders accompanied by sphincter dysfunction (n=1, 4%) such as constipation.

**Table 1:** Intradural Extramedullary Hemangioblastomas (1976-2023).

N	Author, Year	Age (Years)/ Sex	Location	MRI	CTA	AG	PL	ET	Preoperative Symptoms	EOR	Postoperative Outcome
1	Wisoff et al.,1978 (20)	59/M	Conus	No	No	No	Yes	No	Sensory and motor	GTR	Improved
2	Kitanaka et al.,1993 (21)	59/F	T6	No	CT	No	Yes	No	Sensory and motor	GTR	Improved
3	Minami,1998 (22)	48/M	Cervico medullary junction	Yes	No	Yes	Yes	No	Sensory only	GTR	Improved
4	Brisman,2000 (23)	57/F	Conus	Yes	No	Yes	Yes	No	Sensory and motor	GTR	Improved
5	Toyoda et al.,2004 (24)	46/M	C5-C7	Yes	No	No	Yes	No	Sensory only	GTR	Improved
6	Kashiwazaki et al.,2007 (25)	31/M	T4-T6	Yes	No	No	Yes	No	Motor only	GTR	Improved
7	Na et al., 2007 (26)	53/F	C3-C4	Yes	No	No	NS	No	Sensory and motor	GTR	Improved
8		66/M	C7-T1	Yes	No	No	NS	No	Sensory and motor	GTR	Improved
9		35/F	Cervico medullary junction	Yes	No	No	NS	No	Sensory and motor	GTR	Improved
10	Ramachandran et al.,2008 (27)	75/F	C4-C6	Yes	No	No	Autopsy	No	Sensory and motor	GTR	Continued to worsen; death
11	Taniguchi et al.,2009 (29)	65/F	T4-T5	Yes	No	No	Yes	No	Sensory and motor	Marginal resection	Improved
12	Barbosa-Silva et al.,2009 (28)	66/F	T10-T11	Yes	No	No	Yes	No	Sensory and motor	GTR	Improved
13	Chauvet et al.,2014 (30)	51/F	T2、L3、L4	Yes	No	Yes	Yes	No	Sensory only	GTR	Cerebellar hemorrhage associated with acute hydrocephalus; improved
14	Law et al.,2014 (31)	59/M	L4	Yes	CT	Yes	Yes	Yes	Sensory and motor	STR	Improved
15	Blaty et al.,2017 (14)	82/M	L4	Yes	No	No	Yes	No	Sensory only	STR	Improved; temporary urinary and bowel retention
16	Martins et al., 2019 (32)	28/F	L2	Yes	No	No	Yes	No	Sensory only	GTR	Improved
17	Li et al.,2021 (33)	72/M	C5	Yes	Yes	Yes	Yes	No	Sensory only	GTR	Improved
18	Kawanishi et al.,2021 (3)	58/F	T2-T3	Yes	No	No	Yes	No	Sensory and motor	GTR	Improved
19	Sereke et al.,2021 (34)	17/F	T10-T12	Yes	No	No	Yes	No	Sensory, motor and sphincter	GTR	Improved
20	Thippeswamy et al.,2021 (6)	52/F	L1-3	Yes	No	No	Yes	No	Motor only	GTR	Improved
21	Um et al.,2021 (35)	70/F	L2-3	Yes	No	Yes	Yes	Yes	Sensory only	GTR	Improved
22	Shields et al.,2021 (4)	65/M	T12-L1	Yes	CT	No	Yes	No	Sensory only	GTR	Improved
23	Fanous et al.,2022 (37)	62/M	C4-C5	Yes	No	No	Yes	No	Sensory only	GTR	Improved
24	Kehayov et al., 2022 (7)	40/M	Conus	Yes	No	No	Yes	No	Sensory only	GTR	Improved
25	D' Oria S et al.,2022 (36)	49/F	L1/2	Yes	No	Yes	Yes	No	Sensory and motor	GTR	Improved; temporary urinary and bowel retention
26	Fujii et al.,2022 (38)	66/F	L3	Yes	No	Yes	Yes	No	Sensory only	Marginal resection	Improved
27	Present case	53/M	C6-C7	Yes	Yes	Yes	Yes	Yes	Sensory and motor	GTR	Improved

MRI, magnetic resonance imaging; CTA, CT angiography; AG, angiography; PL, pathology; ET, embolization therapy; EOR, extent of resection; M, male; F, female; NS, not specific; GTR, gross total resection; STR, subtotal resection.

**Table 2:** Summary of patient demographics and case characteristics of patient with intradural extramedullary hemangioblastomas.

	Total patients (n=27)	Male (n=12,44.4%)	Female (n=15,55.6%)
**Mean age (range, years)**	54.81 ± 15.33	56.73 ± 14.86	53.00 ± 16.54
Location			
Cervical	6 (22%)	4 (33%)	2 (13%)
Cervicomedullary junction	2 (7%)	1 (8%)	1 (7%)
Thoracic	6 (22%)	1 (8%)	5 (33%)
Lumbar	7 (26%)	2 (17%)	5 (33%)
Conus	3 (11%)	2 (17%)	1 (7%)
Multiple segment	3 (11%)	2 (17%)	1 (7%)
MRI			
Yes	25 (93%)	11 (92%)	14 (93%)
No	2 (7%)	1 (8%)	1 (7%)
CTA			
Yes	2 (7%)	2 (17%)	0 (0%)
No	23 (85%)	8 (67%)	15 (100%)
CT	2 (7%)	2 (27%)	0 (0%)
AG			
Yes	9 (33%)	4 (33%)	5 (33%)
No	18 (67%)	8 (67%)	10 (67%)
PL			
Autopsy	1 (4%)	0 (0%)	1 (7%)
Yes	23 (85%)	11 (92%)	12 (80%)
NS	3 (11%)	1 (8%)	2 (13%)
ET			
Yes	3 (11%)	2 (17%)	1 (7%)
No	24 (89%)	10 (83%)	13 (93%)
EOR			
GTR	23 (85%)	10 (83%)	13 (87%)
STR	2 (7%)	2 (17%)	0 (0%)
Marginal resection	2 (7%)	0 (0%0	2 (13%)
Preoperative Symptoms			
Sensory only	11 (41%)	7 (58%)	4 (27%)
Motor only	2 (7%)	1 (8%)	1 (7%)
Sensory and motor	13 (48%)	4 (33%)	9 (60%)
Sensory, motor and sphincter	1 (4%)	0 (0%)	1 (7%)
Postoperative Outcome			
Improved	23 (85%)	11 (92%)	12 (80%)
Improved; temporary urinary and bowel retention	2 (7%)	1 (8%)	1 (7%)
Cerebellar hemorrhage associated with acute hydrocephalus; improved	1 (4%)	0 (0%)	1 (7%)
Continued to worsen; death	1 (4%)	0 (0%)	1 (7%)

MRI, magnetic resonance imaging; CTA, CT angiography; AG, angiography; PL, pathology; ET, embolization therapy; EOR, extent of resection; GTR, gross total resection; STR, subtotal resection.

MRI is a highly accurate imaging modality for diagnosing spinal hemangioblastoma and has been widely utilized in clinical practice (3, 4, 6, 7, 14, 22–38). Therefore, most patients underwent MRI examination (n=25, 93%), while a few of patients (n=2, 7%) did not undergo such examination. Similarly, only a few of patients underwent CTA or CT, most of patients (n=23, 85%) did not undergo CTA examination. Although spinal angiography was beneficial for differential diagnosis, it was not conducted in over half of the patients (n=9, 33%). Additionally, only 3 patients (including this case) received interventional embolization to occlude the feeding artery.

In the majority of cases, GTR was performed with clear tumor boundaries (n=23, 85%). Only 2 patients underwent marginal resection due to spinal cord adhesion and severe nerve root involvement, while another 2 patients underwent STR. The majority of patients had a favorable postoperative prognosis with recovery of neurological function and resolution of pain or sensory disturbances (n=23, 85%). Transient urinary retention and intestinal retention were observed in only 2 patients after surgery but subsequently improved. Post-surgery, only 1 patient encountered acute cerebellar hemorrhage, while another exhibited progressive sensory and motor function decline, ultimately resulting in death.

Postoperative pathology confirmed 24 cases, 3 cases of IDEM hemangioblastoma that did not yield a clear pathological diagnosis. Only 1 patient’s diagnosis was made through autopsy examination.

## 4 Discussion

Although the clinical symptoms of spinal hemangioblastoma may overlap with those of various spinal degenerative diseases, and the MRI imaging features may resemble a range of tumors and vascular malformations (such as arteriovenous malformations and dural arteriovenous fistulas), MRI examination remains the preferred diagnostic modality for spinal hemangioblastoma (4, 35). The key to MRI diagnosis of hemangioblastoma lies in the identification of abnormally dilated blood vessels located beyond the peritumoral region (24). Due to the location and size of spinal HBs, there may be various imaging manifestations. For instance, smaller lesions typically exhibit solid and homogeneous characteristics with prominent enhancement on T1-weighted images (T1WI), often accompanied by edema and cavity formation on T2-weighted images (T2WI). Conversely, larger lesions demonstrate heterogeneous enhancement on both T1WI and T2WI (6, 13, 26, 39).

Additionally, the pathological diagnosis remains indispensable. Although the histogenesis mechanism of HBs remains elusive, several studies have indicated that HBs demonstrate positive staining for inhibin-α, CD56, and S100, while showing negative staining for CD34 and CD31 (40, 41). Meanwhile, Vimentin, a protein found in intermediate filaments, is expressed within endothelial cells. Ki67, an antigen associated with cellular proliferation, serves as an accurate indicator of tumor cell proliferative activity and is commonly employed for definitive diagnosis (29).

Relevant literature has also indicated that selective spinal angiography demonstrates a sensitivity of up to 90% in the diagnosis of hemangioblastoma (8), which aids in distinguishing it from other tumors and vascular malformations, such as arteriovenous malformations, dural arteriovenous fistulas, and cavernous malformations (10, 42). The utilization of spinal angiography additionally offers comprehensive information regarding the feeding arteries of the lesion, particularly focusing on the anterior spinal arteries, as well as venous drainage. Consequently, this further enhances preoperative preparation (10, 43, 44). In addition, some investigators argue that preoperative embolization can effectively mitigate the risk of intraoperative bleeding (4, 10, 42), while others contend that preoperative embolization does not offer a definitive therapeutic benefit but may potentially incite bleeding or result in medullary infarction during the embolization procedure (45–47). Therefore, it is imperative for personnel performing spinal angiography to possess a high level of technical proficiency and extensive experience.

Due to the rapid deterioration of symptoms in patients with spinal HBs, microsurgical intervention is often necessary. Currently, microsurgery can be classified into GTR and STR based on the extent of resection. Wang et al. (13) suggest that for sporadic or isolated spinal cord HBs, GTR can effectively cure the disease, alleviate clinical symptoms, and have a low recurrence rate. Meanwhile, Imagama S et al. (11) proposed that STR may not effectively address the cavity and edema surrounding hemangioblastoma, potentially exacerbating the patient’s symptoms. The majority of cases advocate a posterior approach for achieving complete tumor resection due to the sufficient space provided by posterior laminectomy, facilitating tumor exposure (48, 49). It is worth noting that despite the limited availability of conclusive evidence, certain scholars contend that stereotactic radiotherapy and antiangiogenic therapy may serve as viable treatment modalities for spinal hemangioblastoma in cases where complete resection is not feasible (14–18). Additionally, it is widely acknowledged among investigators that the prognosis for patients with VHL syndrome is often suboptimal, characterized by a recurrence rate as high as 33.3%. In contrast, sporadic HBs exhibit a lower recurrence rate ranging from 6.25% to 20% (2, 4, 13, 38). Consequently, GTR remains the current preferred treatment for spinal HBs whenever feasible.

The IDEM hemangioblastoma is a rare condition that lacks specific clinical features, but its prominent imaging feature is the vascular flow void effect shown by MRI (39, 50–52). However, it should be noted that arteriovenous malformations and other hypervascular tumors can also exhibit this vascular flow void effect. Therefore, despite completing comprehensive CTA and MRI examinations prior to surgery in our center, it is crucial to differentiate between these two distinct vascular variants - arteriovenous malformation or hemangioblastoma. Furthermore, while DSA currently serves as the most accurate cerebral angiography method with high resolution capabilities, its contribution to the diagnosis of hemangioblastoma still requires evaluation. We firmly believe that pathology remains the gold standard for diagnosing solid tumors due to its ability to assess cellular and molecular landscapes as well as identify benign and malignant tumors.

Up to the present time, the International Society for the Study of Vascular Anomalies (ISSVA) has established a widely acknowledged classification system for vascular anomalies. In recent years, with the flourishing development of genetic testing and imaging technology, there has been a significant enhancement in diagnosing such diseases. Genetic testing has become increasingly comprehensive, aiding in identifying specific genetic mutations and informing treatment decisions. However, when it comes to hemangioblastoma, there still exists an unbridged gap in effective targeted molecule therapy. Furthermore, advanced imaging techniques like MRI and CT have greatly improved our diagnostic capabilities by enabling visualization and detection of vascular abnormalities, thereby creating conditions for achieving more precise diagnoses. Nevertheless, despite advancements in genetic testing and imaging techniques, diagnosis remains challenging due to the rarity of these disorders and their wide range of underlying symptoms. This necessitates a combination of medical resource allocation within the responsible department to minimize risks associated with curative interventions.

After conducting extensive literature analysis, we have observed that a majority of investigators generally advocate for the complete resection of tumors as it effectively reduces tumor recurrence rates. Despite the remarkable outcomes achieved through the application of keyhole minimally invasive techniques in intraspinal space-occupying lesions (53), this particular patient presented with IDEM hemangioblastoma characterized by an abundance of blood vessels and involvement of high segments. Therefore, to ensure surgical safety, a surgical approach involving laminectomy and spinous process resection was chosen subsequent to interventional embolization. Additionally, to ensure spinal stability, we have incorporated the use of commonly employed titanium nails for lamina fixation during vertebroplasty (**Figure 1R**). It is worth mentioning that preoperative imaging examination revealed a significant abundance of tumor vessels within the tumor itself. Consequently, in a hybrid operating room setting, we performed endovascular intervention and successfully occluded over 90% of these tumor vessels using Onyx18, resulting in substantial reduction in intraoperative bleeding.

## 5 Conclusion

Our case illustrates that a combined approach involving endovascular embolization and laminectomy for cervical intramedullary dural HBs, unrelated to VHL syndrome, can effectively minimize intraoperative blood loss, improve surgical field visibility, and enable precise micromanipulation, ensuring complete tumor resection. This comprehensive treatment strategy significantly reduces intraoperative risks and achieves superior therapeutic outcomes. In conclusion, we recommend giving full consideration to preoperative embolization in cases where such procedures are sufficiently qualified. Last but not the least, we advocate for further research to investigate the efficacy of comprehensive treatment strategies for these benign yet high-risk hypervascular tumors.

## Data Availability

The datasets presented in this article are not readily available because of ethical and privacy restrictions. Requests to access the datasets should be directed to the corresponding authors.
